# Physical activity attitudes, intentions and behaviour among 18–25 year olds: A mixed method study

**DOI:** 10.1186/1471-2458-12-640

**Published:** 2012-08-10

**Authors:** Amudha S Poobalan, Lorna S Aucott, Amanda Clarke, W Cairns S Smith

**Affiliations:** 1 Public Health Nutrition Research Group, Division of Applied Health Sciences, University of Aberdeen, Polwarth Building, Foresterhill, Aberdeen, AB25 2ZD, UK; 2 School of Health, Community and Education studies, Northumbria University, Coach Lane Campus, Benton, Newcastle upon Tyne, NE7 7XA, UK

**Keywords:** Physical activity, Young people, 18–25 year olds, Mixed methods, Obesity, Transition

## Abstract

**Background:**

Young people (18–25 years) during the adolescence/adulthood transition are vulnerable to weight gain and notoriously hard to reach. Despite increased levels of overweight/obesity in this age group, physical activity behaviour, a major contributor to obesity, is poorly understood. The purpose of this study was to explore physical activity (PA) behaviour among 18–25 year olds with influential factors including attitudes, motivators and barriers.

**Methods:**

An explanatory mixed method study design, based on health Behaviour Change Theories was used. Those at university/college and in the community, including those Not in Education, Employment or Training (NEET) were included. An initial self reported quantitative questionnaire survey underpinned by the Theory of Planned Behaviour and Social Cognitive Theory was conducted. 1313 questionnaires were analysed. Results from this were incorporated into a qualitative phase also grounded in these theories. Seven focus groups were conducted among similar young people, varying in education and socioeconomic status. Exploratory univariate analysis was followed by multi staged modelling to analyse the quantitative data. ‘Framework Analysis’ was used to analyse the focus groups.

**Results:**

Only 28% of 18–25 year olds achieved recommended levels of PA which decreased with age. Self-reported overweight/obesity prevalence was 22%, increasing with age, particularly in males. Based on the statistical modelling, positive attitudes toward PA were strong predictors of physical activity associated with being physically active and less sedentary. However, strong intentions to do exercise, was not associated with actual behaviour. Interactive discussions through focus groups unravelled attitudes and barriers influencing PA behaviour. Doing PA to feel good and to enjoy themselves was more important for young people than the common assumptions of ‘winning’ and ‘pleasing others’. Further this age group saw traditional health promotion messages as ‘empty’ and ‘fear of their future health’ was not a motivating factor to change current behaviour.

**Conclusion:**

18**–**25 year olds are a difficult group to reach and have low levels of PA. Factors such as, ‘enjoyment’, ‘appearance ‘and ‘feeling good’ were deemed important by this specific age group. A targeted intervention incorporating these crucial elements should be developed to improve and sustain PA levels.

## Background

Young people (18–25 years) in transition from adolescence to adulthood once embarked on independent living are vulnerable to weight gain, that is when they start higher education/employment, living with partners or getting married and/or become parents themselves [1-3]. Reduction in physical activity, changes in dietary pattern (skipping breakfast, eating outside the home), increased social activities all contribute to lifestyle changes making weight gain more likely [4-6]. Individual health behavioural patterns developed during this transition often persist into later life [7] potentially influencing themselves, their partners and/or their children. Between the years 1991 to 2001, the greatest increase in obesity (BMI >30) was amongst the 18–29 year olds rising from 7.1% to 14% [8,9]. Despite these lifestyle changes and the consequent long-term impact on health, this age group are often neglected compared with children or middle aged adults [10,11] possibly because they are hard to reach. Their physical activity (PA) patterns are poorly understood [12] and exploring factors affecting PA behaviour is crucial to developing any intervention hoping to be effective in preventing obesity in this group. Previous studies addressing PA in young people using behavioural theories [13-15] have been conducted either on a wider age group, were focused specifically on university students or based only on quantitative study methodology. This study is one of the first to explore attitudes, intentions and PA behaviour along with related lifestyle factors in this vulnerable age group, and uses a mixed method study design and based on health Behaviour Change Theory.

## Methods

An explanatory mixed method design was used to understand PA behaviour and related lifestyle factors amongst 18–25 year olds living in the Grampian area of North-East of Scotland through a questionnaire survey and focus groups. Explanatory mixed method design is a two phased study, which starts with the collection of quantitative data followed by qualitative data. Qualitative data follows from or connects to the quantitative data and is used to explain or expand on the initial quantitative results.

### Data collection methods

#### Questionnaire survey

Guided by an NHS Grampian steering group, a questionnaire was designed for the quantitative survey based on the Theory of Planned Behaviour (TPB) [16] and Social Cognitive Theory (SCT) [17], both commonly used for health behaviour change. The questionnaire included demographic factors including self reported height and weight; three PA behaviours (active exercise, hours of TV watching and time spent on computer/games console), attitudes, subjective norm, perceived behavioural control (PBC), intention towards PA and barriers and facilitators for achieving recommended levels of PA.

For active PA behaviour, participants were asked on average the number of days per week they would normally be moderately physically active (that is exercise sustained for many minutes, without exhaustion or extreme fatigue that increases the breathing and heart rate, such that the pulse can be felt with increased warmth and possible sweating) as recommended by National guidelines. Further the guidelines suggest that adults should achieve this a minimum of 30 minutes a day on at least five days or more a week for general health benefit [18]. This is the definition used in this study denoted here as being ‘adequately physically active’. Physical activity might include sports, recreational activity and general active living. Those achieving the recommended levels of PA only up to 4 days a week were grouped as being ‘inadequate exercisers’ while those managing this on 5–6 days per week were grouped as ‘adequate exercisers’. Two questions addressed sedentary behaviour. These were the number of hours spent each day watching TV and similarly on computer/games consoles. For each, the response originally had five options but these were compressed into three categories ‘Less than half an hour’, ‘1-4 hours’ and ‘>4 hours’.

Attitudes toward PA were assessed using four concepts – difficult/easy, relaxing/stressful, not enjoyable/enjoyable and unhealthy/healthy. These were assessed by a 5-point scale 1 (disagree) up to 5 (agree) but later regrouped into ‘positive’, ‘neutral and, ‘negative’. A question on PBC asked about the confidence young people had about being moderately physically active. This was coded from 1 (Not very confident) up to 5 (Very confident). The question about young peoples’ intention about being physically active was another 5-point scale, and remained as such, 1 (disagree) up to 5 (agree).

In addressing the facilitators, participants were asked if they would consider doing more exercise for any of 11 reasons given in the question each with a ‘yes/no’ option. Three of the statements related to ‘health’ (improve health, lose weight or maintain healthy weight, and feel fit), one was to improve appearance, three statements referred to relaxing (have fun, socialise, to relax or feel better), one was about competing (to win), two were related to the subjective norm (to please family/friends or to impress) and the last one was ‘others’. Apart from the subjective norm statements, the rest were grouped into four categories: health, appearance, relaxing/socialising and winning.

Similarly, for barriers, the original question had 19 statements, (‘yes/no’ response options), where each statement represented a reason preventing them from taking more exercise. After inspection, these statements were regrouped into 12 barrier classes: PA with the opposite sex; competition; a lack of privacy, information, company, facilities, time and money; having a disability; feeling that they do enough exercise already; bad weather; and finally a poor choice of activities.

These compressed facilitator and barrier classes required revised coding. Classes that combined 3 statements were coded: ‘Strong (facilitator or barrier)’ if all three statements were ‘yes’; ‘Mostly yes’ if two were ‘yes’; ‘Mostly no’ if two were ‘no’ and ‘Not a (facilitator or barrier)’ if all three were ‘no’. Similarly when 2 statements were combined, the coding was revised to: ‘Strong (facilitator or barrier)’ if both statements were ‘Yes’; ‘Not a (facilitator or barrier)’ if they said ‘no’ to both and ‘Mixed’ if they ticked ‘yes’ to one and ‘no’ to the other.

Recruitment of the sample was only possible through an institutional or global approach, since direct access to young people was not permitted. Consequently, the questionnaire was sent electronically via institutes to all university/college students in the Grampian area in 2007–08. They were asked to complete the questionnaire if they were between 18–25 years of age (those not in this range were filtered out). To capture young people not in education, employment or training (NEET), hard copies were sent to co-ordinators of the NEET groups in the Grampian area to be completed by participants at their groups meetings. To capture those at work and young people who may not attend the NEET group sessions, a postal hard copy of the questionnaire was sent to a 2% random sample of 18–25 year olds (n = 1800) in the community using the Community Health Index (CHI), a computer based population index used by NHS Scotland.

#### Focus groups

Using the website for the university, young people between the ages of 18–25 years were invited to take part in focus group discussions using a ‘pop up’ advert. An institutional e-mail with an information letter was also sent to all the students. All the NEET groups and other youth groups/clubs in Grampian area were again approached through the group co-coordinators and given an information letter. Recruitment was also conducted through local media (radio). Seven focus groups resulted with a total of 26 participants from the same population as the quantitative survey. Focus groups gather participants’ attitudes, feelings, beliefs, experiences and reactions in a collective way, not feasible using other methods, for example, observation, one-to-one interviewing or questionnaire surveys [19]. A topic guide, based on issues identified from the survey and grounded in TPB and SCT, facilitated discussion and participants were encouraged also to discuss issues of concern to them, ensuring an inductive approach. Question addressed in the focus group discussions related to actual physical activity behaviour, the importance and perceived relevance of regular exercise at this stage in life and in the future, positive and negative outcome expectations of regular exercise, perceived and actual barriers to undertaking regular exercise, self-efficacy and exercising control over undertaking regular exercise, and finally factors that might facilitate and encourage regular exercising. A purposive sampling method was used based on the previous survey results (age, level of education, employment status) with the intention of obtaining a balance in terms of socio-economic groups. The focus group data collection was terminated after obtaining saturated data from a wide range of relevant groups. A written informed consent was obtained from the participants at the time of the focus groups ensuring anonymity and confidentiality. Ethical approval was obtained from NHS Grampian for the quantitative study and from university ethics committee for the qualitative study.

#### Data analysis methods

For the questionnaire survey, initially, univariate analyses were conducted identifying significant variables, then a multi staged model was developed to associate PA behaviours to the theoretical mediating variables.

#### Exploratory univariate analysis

**Figure 1:**
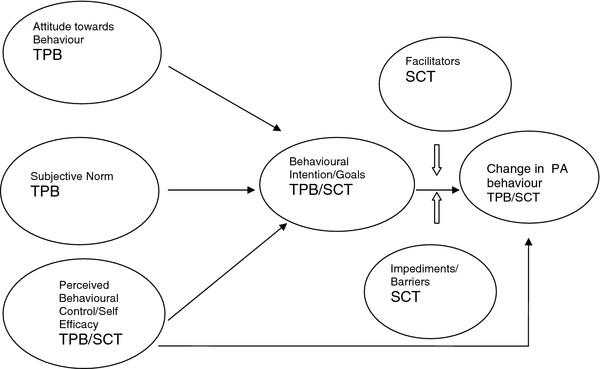
**Constructs of Theory of Planned Behaviour and Social Cognitive Theory.** The main constructs of Theory of Planned Behaviour and Social Cognitive Theory and the linkages.

Frequencies of all the behavioural theory constructs were assessed with demographic factors. The associations between the TPB constructs (attitudes, subjective norm and perceived behavioural control) were assessed with behavioural intention and then with each of the PA behaviours. Similarly the SCT constructs (barriers and facilitators) were analysed with demographics and the PA behaviours. The relationships between the constructs from the two theories are all graphically presented in Figure 1.

#### Multistage modelling

After identifying significant variables from the univariate analyses detailed above, a strategic stepwise methodology was developed for modelling executed in three stages. Initially, behavioural intention was modelled against demographics/risk factors and each TPB construct (attitudes, subjective norm (SN) and perceived behavioural control (PBC)). From this, only significant variables were considered in a Combined Intention Model. Secondly, each PA behaviour was separately modelled with 1) demographics 2) constructs of TPB 3) intention and 4) barriers and facilitators. Finally a combined model was developed using Forward Stepwise Logistic Regression to predict each of the physical activity behaviour, based on only those significant variables from the previous stages. The final model(s) above (one for each PA behaviour) provided the most important associates for PA behaviour.

***‘Framework analysis’*** was used to analyse focus group data in a systematic way [20]. Framework analysis uses a thematic framework to classify and organise data according to *apriori* themes and concepts and also emergent categories from the data. It allows transparent data management and comparison of data between groups. As each group was analysed, themes were added and amended until an agreed framework of themes was developed. Data was therefore explored within a common framework that was both grounded in the theory and informed by participants’ views and experiences.

After analysing the quantitative and qualitative data separately using their respective appropriate analytical approaches, a ‘*side-by-side comparison’*, method was used. This enabled the comparisons and synthesis of the results from both quantitative and qualitative components [21].

### Results of questionnaire survey

#### Exploratory and univariate analysis

##### Physical activity (PA) behaviour

1313 completed questionnaires, representing 4% of the 18–25 years olds living in the Grampian area, were analysed (1029 from university/colleges and 284 from the community). Only 28.1% were adequately ‘physically active’ with 68.6% watching TV for >4 hours/day and 57.7% being on computer/games console for >4 hours/day.

##### Attitudes, subjective norm, perceived behavioural control and intentions towards PA

Despite 66% considering regular exercise to be healthy, only around 20-30% found exercise easy, relaxing and enjoyable (Additional file 1: Table S1). In fact, around 13% and 6% felt exercise to be difficult and stressful respectively. Surprisingly, only 34% felt it important to please and/or impress others (subjective norm). While 81.5% intended to do adequate exercise, only 59% were reasonably confident about being active.

##### Associations between the individual constructs and demographic factors

Health outcomes/constructs had similar univariate associations with age as with levels of education (Additional file 2: Table S2). Those older (23+ vs 18-19) tended to be heavier with lower levels of PA, spent longer on computer/games and did not find PA relaxing or enjoyable. Postgraduates, in particular spent more time on computer/games. The younger age groups needed to please others more. With respect to gender, males did more exercise, were more confident about exercising and found PA easy, enjoyable and relaxing. Males also exercised more to please others. Despite this, men were heavier and spent more time watching TV/game consoles. Compared with students studying ‘other’ subjects, those studying health related subjects were generally more active, felt strongly that doing PA was ‘healthier’ but had the need to please others. Science students, compared with students doing ‘other’ courses, whilst having strong intentions to do adequate amounts of exercise were not confident they could. Heavy smokers, unemployed and/or ill tended to be more obese and sedentary. They also found PA difficult, stressful, not enjoyable and had less intention of doing adequate amounts. Enough exercise was also difficult and stressful for those living alone who lacked confidence that they could do sufficient exercise.

##### Association between the individual constructs

Young people with positive attitudes towards exercise had strong intentions and, were confident they could do sufficient amounts of PA (Additional file 3: Table S3). Of those who found PA difficult and stressful about half still had strong intentions. Those with little intention to do exercise not only found PA difficult, stressful and unenjoyable but also tended to be sedentary. With respect to actual behaviour, those doing sufficient exercise generally had strong intentions but 57% of those not doing adequate PA still had high intentions to do so. Although the subjective norm (do PA to please others) seemed important for the youngest age group, it was not associated either with PA intention or behaviour.

##### Association between PA behaviours and Body Mass Index (BMI)

BMI was significantly associated with all three physical activity behaviours (Additional file 4: Table S4). Those doing more exercise were less overweight/obese; sedentary behaviour (TV watching/console gaming) was associated with higher weights, particularly if this exceeded more than 4 hours/day.

#### Multistage Modelling

##### Stage 1

Intention to do adequate amounts of exercise (Additional file 5: Table S5) was associated with employment status, attitudes (PA being ‘easy/difficult’, ‘enjoyable’ and ‘healthy’) and PBC but not with the subjective norm. When these blocks were combined, all remained significant explaining around 50% of the overall variation in this intention construct (R^2^ =0.552).

##### Stages 2 and 3

**Active Behaviour (AB)** (reduced to ‘do enough’ vs ‘don’t do enough’): For Stage 2, Active Behaviour was modelled against the blocks of variables: demographics, attitude, intention, PBC, subjective norm, facilitators and barriers. From each, AB was associated with (Additional file 6: Table S6) gender and BMI, an attitude (PA ‘easy/difficult’), perceived behavioural control, an intention (to be adequately active), a facilitator (wanting to win) and several barriers (‘lack of choices’, ‘already doing enough exercise’ and ‘time’). The Stage 3 Active Behaviour model, combined significant variables from Stage 1 and each Stage 2 (AB) models. Only PA being easy/difficult’, ‘PBC’ and the barrier ‘already do enough exercise’ remained significant (R^2^ = 0.523). This indicates that those doing insufficient exercise (79%) were likely to find exercise difficult, while those who perceived they had control over their behaviour were confident that they could be active and probably were already doing enough.

##### TV sedentary behaviour (TV)

Stage 2 model (TV) (Additional file 6: Table S6) had significant associations with demographic variables (employment status and BMI categories), attitudes (PA ‘easy/difficult’ and ‘not enjoyable’) and intention but not with PBC or the subjective norm. One facilitator (PA helps you to relax) and several barriers (disability, lack of choices and bad weather) were also significant. The Stage 3 TV Sedentary Behavioural model incorporating the important variables from each of the Stage 2 (TV) models and Intention variables from Stage 1, did not have a good fit (R^2^ =0.081). There is a hint that more TV watching hours was associated with being heavier, having little intention of doing more PA and the perception that there was a lack of choice in activities.

##### Computer/games sedentary behaviour (Comp)

Stage 2 model for this sedentary behaviour (Additional file 6: Table S6) had associations with demographics (gender, level of education), attitudes (PA ‘enjoyable’) but also with having a disability. Neither PBC, physical activity intention nor the subjective norms were associated with this sedentary behaviour. When combined in a Stage 3 (combining Stage 1 and each Stage 2 (Comp) variables), the full computer/games Sedentary behaviour model (R^2^ =0.065) had only two significant variables (gender and level of education) again tentatively hinting that those on computer/games for more than four hours were more likely to be males and postgraduates. Also included in the final model was if the participant had a disability that would prevent them from doing more exercise. While an obvious barrier, it is surprising that it was included in the model given that it represented only 5% of the sample.

The physical activity behaviour among 18–25 year olds and the relationship with their demographics, attitudes, subjective norm, PBC, intentions including the barriers and facilitators are summarised and presented in Table 1.

**Table 1:** Combined physical activity behaviour model (Logistic Regression Model)

**PA behaviours**	**Attitudes**	**Subjective Norm**	**PBC**	**Behaviour intention**	**Demographics**	**Barrier**	**Facilitator**	**Combined Nag R**^**2**^
***Active exercise***	PA Difficult/Easy	NS	Good PBC	NS	NS	Already do enough exercise	NS	0.523
***TV sedentary***	NS	NS	NS	Strong Intention	Higher BMI	Lack of Choices	NS	0.081
***Computer/games sedentary***	NS	NS	NS	NS	Gender, Level of education	Disability	NS	0.065

PA: Physical Activity; PBC: Perceived Behavioural Control; NS: Not Significant.

Nag R^2^: Nagelkerke R squared – pseudo measure of fit.

### Results of focus groups

Seven focus groups were conducted and the characteristics are presented in Table 2.

**Table 2:** Characteristics of the focus groups

**Focus group**	**Code**	**Characteristics**	**No of participants (M/F)**	**Mean age (range)**
University	T0	Older group	5 (1/4)	22 (20–24)
University	C0	Younger group	8 (3/5)	19 (18–19)
College	V0	Working/training 1	2 (0/2)	21 (20–22)
College	M0	Working/training 2	2 (1/1)	20 (18–21)
Inner City	H0	Young mothers	3 (0/3)	23 (21–24)
Inner City	P0	Mixture of working/not working	4 (0/4)	19 (18–21)
Shire (Rural area)	K0	Community Youth group- not in education or employment	2 (1/1)	19 (18–19)

Five themes were identified from the focus group discussions: physical activity behaviour, influences on PA behaviour, attitudes, behaviour change and knowledge. Within each theme, several subthemes were identified (Additional file 7: Table S7).

#### Present and past PA behaviour

Physical activity level among 18–25 year olds varied widely, with older (aged 20+) participants studying at university/college reporting doing more vigorous activities (kite surfing, mountain biking and martial arts) compared with the other groups (Additional file 7: Table S7, quote 1a and b). The main ‘other’ forms of physical activity were walking to places, looking after children and that undertaken during the course of paid employment (Additional file 7: Table S7, quote 1c and d). Irrespective of the groups, all young people felt that their levels of activity had decreased as they got older (Additional file 7: Table S7, quote 1e and f).

#### Influences on PA behaviour

While being exposed to PA at a young age by parents, observing their fitness and being encouraged helped participants in university/college to be physically active, necessity was a reason for some (Additional file 7: Table S7, quote 2a and 2b). Other reasons for exercising were to promote feelings of enjoyment, well being and having confidence to be physically able and to counter feelings of ‘depression’ and ‘grumpiness’ (Additional file 7: Table S7, quote 2c to 2f). Negative influences about PA behaviour included ‘student lifestyle’, lack of company, time and cost restrictions (Additional file 7: Table S7, quote 2g to 2i). Lack of facilities, (equipment in gyms leading to long queues) and lack of privacy (at swimming pools) was also highlighted (Additional file 7: Table S7, quote 2j). Young people reported that there were not adequate facilities conducive for this age group to be active and that they felt neglected by society (Additional file 7: Table S7, quote 2k). Competitive sports generally had a negative influence on both younger people at university and those in inner city areas (Additional file 7: Table S7, quote 2l). Some other negative influences reported included laziness, not being bothered, bad weather and safety concerns (Additional file 7: Table S7, quote 2m to 2o). Other factors influencing behaviour were the assumption that exercising took a lot of effort (Additional file 7, quote 2r) and only working out in the gym/participating in organised sports counted as ‘good’ physical activity while walking and active living did not (Additional file 7: Table S7, quote 2p and 2q). There was no strong evidence that participants did any form of exercise to please others. In fact, university students resisted the pressure to imitate celebrities but were keen to exercise for their own benefit (Additional file 7: Table S7, quote 2s).

#### Attitudes towards PA

Participants preferred doing PA for enjoyment rather than meeting social expectations. In spite of highlighting the lack of facilities in gyms, they preferred walking, to other exercise. Young mums felt that young children could be included in walks and there was no cost involved. Those who preferred the gym, felt it was the most convenient and easiest way to do exercise requiring less planning or organisation than other forms of exercise. University students felt that cutting down on one type of exercise to undertake another type of exercise was inappropriate; for example, taking the bus to go to the gym rather than ‘walking’ (Additional file 7: Table S7, quote 3a). College students felt it was hard to continue with the same exercise for a long time and reported phases of not exercising. Those who did adequate amounts of physical activity felt that their friends focused too much on diet rather than exercising for a healthy lifestyle (Additional file 7: Table S7, quote 3b). University students felt that some peers had negative attitudes towards regularly exercising and that people would not help or join them (Additional file 7: Table S7, quote 3c). In spite of some positive influence from parents, participants from the inner city groups were not keen to respond to offers made by their mothers to exercise together (Additional file 7: Table S7, quote 3d). Generally, although keen to stay healthy and be physically able when they got older, participants were unworried about putting on weight and/or did not think that far into the future (Additional file 7: Table S7, quote 3e and 3f). Some believed existing information was inadequate and ambiguous. Health messages were seen as *'empty information'* providing broad facts about health without detail. They also felt they did not focus on the right message for young people. Participants felt that messages such as *‘sport is fun’* would encourage them to pursue regular exercise.

#### Behaviour change

Company and/or encouragement from friends and partners were identified as motivating factors to increase PA by all groups (Additional file 7: Table S7, quote 4a). Non-competitive sports/activities, good publicity of sport clubs/activities would motivate university students. In comparison, inner city participants identified facilities tailored for their age group with subsidised fees, setting goals to achieve targets and group discussion on health as motivating factors for more exercise (Additional file 7: Table S7, quote 4b and 4c). There was strong intention to do more exercise across all groups but no evidence of perceived control of behaviour. Intentions were often not translated into action for many participants (Additional file 7: Table S7, quote 4d and 4e).

#### Knowledge

Across all groups, participants were aware of the benefits of exercise and the consequences of doing less PA. Despite this, university participants felt that they needed educating on the types of exercises and the benefits of each (Additional file 7: Table S7, quote 5a and 5b). Based on the knowledge they had, a few participants (from college and the inner city group) tried to motivate others to exercise without much success (Additional file 7: Table S7, quotes 5c and 5d).

## Discussion

This study explored the physical activity behaviour and influencing factors in this vulnerable and hard to reach age group. Included were, not only 18–25 year olds from university and colleges but also those who were working and those not in education, employment or training (NEET groups). The mixed method study design identified factors affecting behaviour and unravelled details of these and other factors affecting behaviour through interactive focus groups discussion. This study showed that only 28% of 18–25 year olds achieved adequate physical activity levels as recommended by national guidelines, lower than reported for 2000 in England (58% of men and 32% of women amongst 16–24 year olds)[22]. PA levels here decreased with age and the time spent on computers/game consoles, whether for work/study or pleasure, increased gradually within this age group (12% to 20%).

In this study, positive attitudes (PA easy to do) was associated with being active and reduced TV watching. A recent review looking at descriptions by 11–16 year old girls of what it meant to *‘become a woman’* suggests that PA participation was *‘babyish’ *[23]. This attitude may be contributing to the decreased levels of physical activity among 18–25 year old females in this study. There exists an attitude that changing diet behaviour was easier than exercising [24] and that only a gym work out/participate in organised sports counted as ‘real’ exercise. The perception that they already undertook enough exercise and did not need to do more might stem from the attitude/belief that they might not become obese[25]. The subjective norm variable did not predict any PA behaviours while young people will participate in activities for fun rather than to win or impress other people. PBC was associated with the final of the active exercise model but not with the sedentary behaviours. The focus groups revealed that many did not commence any new sport after moving to university/job or having children, in spite of being active at school. This could be because PA is more ‘organised’ in schools and becomes an individual’s responsibility once they become independent.

Despite good intentions to do more exercise, young people were unable to translate these into actual behaviour. While employment status, positive attitudes with PBC explained 55% of the physical activity intention, translation of intention into behaviour was poor; intention itself only explained 5.7% of the active exercise behaviour, 3% of sedentary TV watching behaviour; was non-significant for sedentary computes/games behaviour (Additional file 6: Table S6) and remained significant in only TV watching once other variables were considered.

Barriers for doing adequate amounts of exercise identified from the survey were lack choices of activities and disability. However, focus groups identified specific issues to this age group. Although competitive sports and winning was identified as a motivator in the survey, particularly for men, it was seen as a major discouragement for many in the (female dominated) focus group discussions. Inadequacy and low self-esteem regarding body image made going to a gym or swimming pool, with the opposite sex, a barrier for females, mainly single mums. Studies have shown that those with low competence and self esteem do not generally engage in physical activity [12]. Hence, improved facilities and activities focussed on single sex could motivate young women of this age group to participate more in physical activity.

Young people, although aware of the consequences, had no concerns about their future health. Obesity and other morbidities, are delayed consequences of a sedentary lifestyle[26] and as such there is no *‘fear factor’* to encourage young people to change their behaviour. Concern for future health, depicted in many of these *‘empty’* health messages, seemed irrelevant to these young people and hence not the necessary concepts to motivate them to be more active.

Several studies in the past have assessed PA based on behavioural theories and found similar results although the strength of the relationships varied across studies [13-15,27-29]. The main strength of the present study is that it captures a vulnerable age group (18–25 year olds) using a wide sample including not only students but also those who worked and those not in education, employment or training. This study also explained the in-depth meaning of the constructs through dynamic and interactive focus group discussions, providing a better understanding of elements relevant for young people.

However, there are several limitations that need to be acknowledged while interpreting the results. Data was collected using a self reported questionnaire with no objective measures of PA behaviour and hence a subject could over/underestimate their behaviour. Although the data could represent typical Caucasian young people undergoing similar transition, this data collection was restricted to a particular part of Scotland and thus will be limited when extrapolated to young people from other cultures especially with respect to the facilitators and barriers. Although efforts were made to recruit young people from the community, either working full time or Not in Employment, Education or in Training (NEET) for both quantitative and qualitative components of the study, this sample was over represented by students and the interpretation of the results should take this into consideration. In addition, for both quantitative and qualitative aspects, recruitment of young people at university/college was only possible through the institutions since direct access to students was not permitted. Major employers denied direct access to young people in work places due to time constraints and data protection issues. Consequently, only a random sample from the community was possible in order to capture those at work. This highlights the recruitment issues in this age group another potential limitation in generalising the results to those who work. It was impossible to calculate the response rate for the questionnaire survey in this study due to the institutional approach and subsequent lack of denominator. Consequently despite the large sample size, this survey only captured approximately 4% of the 18–25 year olds in the Grampian region, as estimated from the census data. For the qualitative study, recruitment was also a major restriction despite diligent attempts. While sufficient for this methodology, only 26 people participated in the qualitative study.

Interventions to improve physical activity in this age group might be successful in some targeted motivated populations [30]. However, replicating these interventions at community level is unlikely to succeed as only a fraction of young people will participate and among those, few will lose weight. It is crucial to address barriers specific to young people and so build on factors motivating them to participate in interventions to improve and sustain their PA levels. For any intervention to be effective, the initial step would be to engage young people to participate and take ownership. Consequently, the traditional health promotion messages deemed ‘empty’ and ‘irrelevant’ by these young people need to be translated or tailored to be more attractive for recruiting and retaining them. Factors pivotal in sustaining such an interest in young people are ‘enjoyment’, 'appearance' and ‘feeling good’. Interventions incorporating these elements are more likely to encourage them to be involved in programmes initiated to bring about behavioural change to improve physical activity. However in the current obesogenic environment, individual responsibility can only be successful along with better provision to healthy lifestyle options [31]. This suggests government, private and voluntary sectors work together to change the societal and environmental factors, whilst supporting individuals who want to make healthy choices[8,32,33]. Future research should involve young people to identify these intervention components.

## Conclusion

18–25 year olds have low levels of physical activity and consequently are vulnerable to weight gain but difficult to reach. A targeted approach as identified in this study might be a starting point to improve PA levels and promote healthy living in this vulnerable age group. This mixed method study identified elements deemed important by this specific group of young people (‘enjoyment’, ‘appearance’ and ‘feeling good’). A targeted intervention should be developed incorporating the crucial elements identified by this age group.

## Abbreviations

BMI: Body Mass Index; CHI: Community Health Index; NEET: Not in Education, Employment or Training; NHS: National Health Service; PA: Physical Activity; PBC: Perceived Behavioural Control; SCT: Social Cognitive Theory; SN: Subjective Norm; TPB: Theory of Planned Behaviour.

## Competing interests

The authors declare that they have no financial or non-financial competing interests.

## Authors’ contributions

AP reviewed the literature, carried out data collection, analysed the data, drafted and revised the manuscript. LA performed the statistical modelling and critically revised the manuscript. AC assisted in the analysis of the qualitative data and critically revised the manuscript. WCSS conceptualised the project and critically revised the manuscript. All authors read and approved the final manuscript.

## Pre-publication history

The pre-publication history for this paper can be accessed here:

http://www.biomedcentral.com/1471-2458/12/640/prepub

## Supplementary Material

Additional file 1 **Frequencies of the Health outcomes and Theoretical constructs.** Frequencies of the Health outcomes and the mediating theoretical constructs of TPB. Click here for file

Additional file 2 **Association between health outcomes and TPB constructs with demographics.** Association between health outcomes and mediating constructs of TPB with demographics. Click here for file

Additional file 3 **Association between TPB constructs and physical activity behaviour.** Association between TPB constructs (Attitudes, SN, PBC, intention) and physical activity behaviour. Click here for file

Additional file 4 **Association between physical activity behaviour and BMI.** Association between the three physical activity behaviours and BMI. Click here for file

Additional file 5 **Physical activity intention model.** Physical activity intention model (Stage 1). Click here for file

Additional file 6 **Physical activity behaviour model.** Physical activity behaviour model (Stage 2 and 3). Click here for file

Additional file 7 Results of the focus groups. Click here for file

## References

[B1] Anderson DA Shapiro JR Lundgren JD The freshman year of college as a critical period of weight gain: an initial evaluation Eat 2003 4 367 10.1016/S1471-0153(03)00030-8 15000962

[B2] Burke V Beilin LJ Dunbar D Kevan M Changes in health-related behaviours and cardiovascular risk factors in young adults: associations with living with a partner Prev Med 2004 39 722 730 10.1016/j.ypmed.2004.02.038 15351538

[B3] Graham MA Jones AL Freshman 15: valid theory or harmful myth? J Am Coll Health 2002 50 171 173 10.1080/07448480209596023 11910950

[B4] Huffman L West DS Readiness to change sugar sweetened beverage intake among college students Eat 2007 8 10 14 10.1016/j.eatbeh.2006.04.005 17174846

[B5] Niemeier HM Raynor HA Lloyd-Richardson EE Rogers ML Wing RR Fast food consumption and breakfast skipping: predictors of weight gain from adolescence to adulthood in a nationally representative sample J Adolesc Health 2006 39 842 849 10.1016/j.jadohealth.2006.07.001 17116514

[B6] Sheehan TJ DuBrava S DeChello LM Fang Z Rates of weight change for black and white Americans over a twenty year period Int J Obes Relat Metab Disord 2003 27 498 504 10.1038/sj.ijo.0802263 12664083

[B7] Parcel G Muraskin L Endert C Community education study report J Adolesc Health Care 1988 9 415 453 10.1016/0197-0070(88)90008-3 3182377

[B8] Huang TT Harris KJ Lee RE Nazir N Born W Kaur H Assessing overweight, obesity, diet, and physical activity in college students J Am Coll Health 2003 52 83 86 10.1080/07448480309595728 14765762

[B9] Mokdad AH Ford ES Bowman BA Dietz WH Vinicor F Bales VS Marks JS Prevalence of obesity, diabetes, and obesity-related health risk factors, 2001 JAMA 2003 289 76 79 10.1001/jama.289.1.76 12503980

[B10] Howarth C Street C Sidelined: Young adults' access to services 2000 New Policy Institute, London

[B11] The Prince's Trust Reaching the hardest to reach 2004 Prince's Trust, London

[B12] Fox KR Hillsdon M Physical activity and obesity Obes Rev 2007 8 115 121 10.1111/j.1467-789X.2007.00329.x 17316313

[B13] Bozionelos G Bennett P The Theory of Planned Behaviour as predictor of exercise: The moderating influence of beliefs and personality variables J Health Psychol 1999 4 517 529 10.1177/135910539900400406 22021644

[B14] Caperchione CM Duncan MJ Mummery K Steele R Schofield G Mediating relationship between body mass index and direct measures of the Theory of Planned Behaviour on physical activity intention Psychol Health Med 2008 13 168 179 10.1080/13548500701426737 18350461

[B15] Wallace LS Buckworth J Kirby TE Sherman WM Characteristics of exercise behaviour among college students: application of social cognitive theory to predicting stage of change Prev Med 2000 31 494 505 10.1006/pmed.2000.0736 11071829

[B16] Nutbeam D Harris E Theory in a nutshell: A guide to Health Promotion theory 1999 McGraw-Hill Book company, Australia

[B17] Bandura A Health Promotion by Social Cognitive Means Health Educ Behav 2004 31 143 164 10.1177/1090198104263660 15090118

[B18] Department of Health Riddoch C, Fox KR Evidence on the impact of physical activity and its relationship to health 2004 Department of Health, London

[B19] Gibbs A Focus groups Social Research Update (19) 1997 Department of Sociology, University of Surrey, Guildford, England

[B20] Ritchie J Spencer L O'Connor W Ritchie J, Lewis J Qualitative Research Practice: a guide for Social Science Students and Researchers Carrying out Qualitative analysis 2005 4 SAGE Publications, London 219 262

[B21] Creswell JW Clark VLP Creswell JW, Clark VLP Designing and conducting mixed methods research Analysing and interpreting data in mixed methods research 2011 2 Sage publications, California 203 250

[B22] Department of Health The health of young people 1995-97 Health Survey for England 2000 Department of Health, London

[B23] Rees R Kavanagh J Harden A Shepherd J Brunton G Oliver S Oakley A Young people and physical activity: a systematic review matching their views to effective interventions Health Educ Res 2006 21 806 825 [Review] [46 refs] 10.1093/her/cyl120 17041020

[B24] Okonkwo O While A University students' views of obesity and weight management strategies Health Educ J 2010 69 192 199 10.1177/0017896910363147

[B25] Mullaney MI Corish CA Loxley A Exploring the nutrition and lifestyle knowledge, attitudes and behaviour of student home economics teachers: baseline findings from a 4-year longitudinal study Int J Consum Stud 2008 32 314 322 10.1111/j.1470-6431.2007.00650.x

[B26] Hill JO Wyatt HR Reed GW Peters JC Obesity and the environment: where do we go from here? Sci 2003 299 853 855 10.1126/science.1079857 12574618

[B27] Armitage CJ Conner M Efficacy of the Theory of Planned Behaviour: a meta-analytic review Br J Soc Psychol 2001 40 471 499 10.1348/014466601164939 11795063

[B28] Boudreau F Godin G Using Theory of planned behaviour to predict exercise intention in obese adults Can J Nurs Res 2007 39 112 125 17679588

[B29] Gardner RE Hausenblas HA Exercise and diet determinants of overweight women participating in an exercise and diet programme: a prospective examination of the theory of planned Behaviour Women Health 2005 42 37 62 16782675 10.1300/j013v42n04_03

[B30] Poobalan AS Aucott LS Precious E Crombie IK Smith WC Weight loss interventions in young people (18 to 25 year olds): a systematic review Obes Rev Obes Rev 2010 11 580 592 [Review] [44 refs] 19874531 10.1111/j.1467-789X.2009.00673.x

[B31] WHO Fact sheet.Obesity and overweight 2011

[B32] Swinburn B Gill T Kumanyika S Obesity prevention: a proposed framework for translating evidence into action Obes Rev 2005 6 23 33 10.1111/j.1467-789X.2005.00184.x 15655036

[B33] Yach D McKee M Lopez AD Novotny T Improving diet and physical activity: 12 lessons from controlling tobacco smoking BMJ 2005 330 898 900 [Review] [10 refs] 10.1136/bmj.330.7496.898 15831879 PMC556169

