# Supplementary material for: Physical activity attitudes, intentions and behaviour among 18–25 year olds: A mixed method study
Source: BMC Public Health. 2012 Aug 10;12:640. doi: 10.1186/1471-2458-12-640 (PMC3490897; doi:10.1186/1471-2458-12-640)
Supplement: Additional file 1 — Frequencies of the Health outcomes and Theoretical constructs. Frequencies of the Health outcomes and the mediating theoretical constructs of TPB. [file 1471-2458-12-640-S1.doc]

Additional file 1: Frequencies of the Health outcomes and the mediating theoretical constructs from TPB

| **Health outcomes and theoretical constructs** | **Frequency** | **Percentages** |
| --- | --- | --- |
| ***Physical activity behaviour*** | | |
| *Active behaviour - exercise*  Adequate exercise  Inadequate exercise | 369  933 | 28.1%  79.1% |
| *Sedentary behaviour-TV watching*  Less than half an hour a day  1 to 4 hours a day  More than 4 hours a day | 408  802  99 | 31.1%  61.1%  7.5% |
| *Sedentary behaviour- computer or games console*  Less than half an hour a day  1 to 4 hours a day  More than 4 hours a day | 548  575  182 | 41.7%  43.8%  13.9% |
| ***Attitude*** | | |
| 1 (Difficult)  2  3  4  5 (Easy) | 165  233  359  234  306 | 12.6%  17.7%  27.3%  17.8%  23.3% |
| 1 (Relaxing)  2  3  4  5 (Stressful) | 261  328  465  163  76 | 19.9%  25.0%  35.4%  12.4%  5.8% |
| 1 (Not enjoyable)  2  3  4  5 (Enjoy) | 56  135  358  350  394 | 4.3%  10.3%  27.3%  26.7%  30.0% |
| 1 (Unhealthy)  2  3  4  5 (Healthy) | 11  21  111  283  868 | 0.8%  1.6%  8.5%  21.6%  66.1% |
| ***Subjective norm*** | | |
| Important  Mixed  Not important | 200  251  829 | 15.2%  19.1%  63.1% |
| ***Perceived Behavioural Control*** | | |
| 1 (No confidence)  2  3  4  5 (High confidence) | 119  152  254  336  439 | 9.1%  11.6%  19.3%  25.6%  33.4% |
| ***Intention*** | | |
| 1 (No intention)  2  3  4  5 (Strong Intention) | 30  55  149  227  841 | 2.3%  4.2%  11.3%  17.3%  64.1% |

TPB: Theory of Planned Behaviour; percentages do not add up to 100% due to missing values
