# Supplementary material for: Physical activity attitudes, intentions and behaviour among 18–25 year olds: A mixed method study
Source: BMC Public Health. 2012 Aug 10;12:640. doi: 10.1186/1471-2458-12-640 (PMC3490897; doi:10.1186/1471-2458-12-640)
Supplement: Additional file 2 — Association between health outcomes and TPB constructs with demographics. Association between health outcomes and mediating constructs of TPB with demographics. [file 1471-2458-12-640-S2.doc]

Additional file 2: Association between health outcomes and TPB constructs with demographics

| ***HEALTH OUTCOMES AND CONSTRUCTS*** | ***DEMOGRAPHIC AND LIFESTYLE FACTORS*** | | | | | | | |
| --- | --- | --- | --- | --- | --- | --- | --- | --- |
|  | **Age** | **Level of education** | **Gender** | **Study subject** | **Employ/study status** | **Smoking** | **Living arrange**  **ment** | **Alcohol** |
| **BODY MASS INDEX (BMI)**  *WHO categories* | **OW/Obesity higher in 23+ (31.9 % vs 21.8% in 18-19 yrs)** | **OW/Obesity higher in PG (33% vs 26% in UG)** | **Obesity higher in Males (9.4% vs 6.8% in F)** |  | **Obesity higher in ill and /or unemployed: (16.3%)**  **Low in employed: (5.6%)** | # Increased trend  of obesity in heavy smk, (9.2% vs 6.9% in NS) |  |  |
| **PA BEHAVIOUR**  Active Exercise | # Decreased levels in 23+ (30.4% in 18-19 yrs vs 24.9% in 23+) |  | **Higher in Males (38.7% vs 24.8% in F)** | **Higher in health students - (36.6% vs 23.7% in arts)** |  |  |  |  |
| >4 hours/day of TV watching |  | **Higher in Foundation level (9.2% vs 4.5% in UG)** | **Higher in Males(9.3% vs 6.8%in F)** | **Less in health students - (4.6% vs 8.4% among arts students)** | **- Higher in ill/unemployed (20.8% vs 6% in students)**  **- More employed watch 1-4 hrs TV (80%)** | **High in heavy smk (12.5% vs 6.4% in NS)** |  |  |
| >4 hours/day on Computer/  game console | **Higher in 23+**  **(20.2 % in 23+ vs 12.6% in 18-19 yrs)** | **Higher in post graduates (26.7% vs 9.1% in UG)** | **Higher in Males (20.2% vs 11.5 % in F)** | **Less in health students - (9.2% vs 16.6% in science)** | **- Higher in ill/unemployed (24.5% vs 11% in employed)** | **Less in heavy smk - (10.1% vs 16.3% in mod smk )** |  |  |
| **PA ATTITUDES**  Difficult/easy |  |  | **Males- Easy**  **( 38.3% vs 18.2 % in F)** |  | **-Employed: easy (32.3%)**  **-Unemployed: difficult (21.6%)** |  | **Alone – difficult**  **(19.7% vs 11.9% with others)** |  |
| Relaxing/  stressful | **Relaxing for 18 -19 yr olds (23.6% vs 19.5% in 23+)** | **Less stressful for UG (2.6% stressed vs 9.5% stressed in PG)** | **Relaxing for Males (27.7% vs 17.4% in F)** |  |  | **Stressful for heavy smk- (11.4% vs 5.1% in NS)** | **Stressful in living alone (13.3% vs 4.7% with others)** |  |
| Not enjoyable/  enjoyable | **Enjoyable for 18 -19 yr olds (32% vs 27.3% in 23+)** | **Enjoyable for UG (33.3% vs 21.1% in PG)** | **Enjoyable for Males (38.6% vs 27.5% in F)** |  | **Enjoyable for students (32.3%)**  **Not enjoyable for Unemployed (11.8%)** | **Not enjoyable for heavy smk (8.2% vs 2.1% of mod smk)** |  |  |
| Unhealthy/  healthy |  |  |  | **Healthy in Health students (69.9% vs 64.8% in arts)** |  | **Healthy in mod smk (73.6% vs 55.4% in heavy smk)** |  |  |
| **PA SUBJECTIVE NORM (Pleasing others)** | **Important for 18-19 yrs (20.6% vs 10.4% in 23+)** | **Important for Foundation level (19.0% vs 12.6% in PG)** | **important for Males (20.1% vs 14% in F)** | **Important for health students (21.3% vs11.6% in arts)** |  | **Less important for NS (imp for 13.9% vs 20.6% in heavy smk)** |  |  |
| **PA PBC (Control over their behaviour)** |  |  | **Males more confident (46.6% vs 29.2% in F)** | **Science students less confident ( 25.6 % confident vs 36.6% confident in others)** | **Employed more**  **confident (59.5% vs 28% in student and employed)** |  | **Living alone less confident (11.9% vs 6.7% in alone Mon-Fri)** |  |
| **PA INTENTION** |  |  |  | **Strong intention in science students (74.8% vs 62.6% in others)** | **Strong intention among students (70.6% vs 37.3% in ill/unemployed)** | **No intention in heavy smk (5.6% had no intention vs 1.6% in NS)** |  | **No intention in medium alcohol (1.6% had no intention vs 2.5% in low alcohols)** |

Significant associations are **BOLDED**; # Statistically non-significant but there was a trend; TPB: Theory of Planned Behaviour; PBC: Perceived Behavioural Control; PA: Physical Activity; F: Females; PG: Post graduates; UG: Under graduates; smk: smokers; NS: non-smokers; Mod smk: 1-5 cigarettes a day; Heavy smk: >5 cigerattes a day
