# Supplementary material for: Physical activity attitudes, intentions and behaviour among 18–25 year olds: A mixed method study
Source: BMC Public Health. 2012 Aug 10;12:640. doi: 10.1186/1471-2458-12-640 (PMC3490897; doi:10.1186/1471-2458-12-640)
Supplement: Additional file 3 — Association between TPB constructs and physical activity behaviour. Association between TPB constructs (Attitudes, SN, PBC, intention) and physical activity behaviour. [file 1471-2458-12-640-S3.doc]

Additional file 3: Association between TPB constructs and physical activity behaviour

| ***TPB CONSTRUCTS*** | ***PA INTENTION*** | ***PA BEHAVIOUR*** | | |
| --- | --- | --- | --- | --- |
| **PA ATTITUDES** |  | Active exercise | TV watching | Computer/games |
| Difficult/easy | **Easy had strong intentions (87.2% easy vs 52.1% difficult)** | **Adequate exercise found easy (59.1% easy vs 1.1% difficult)** | **Difficult ;watched >4 hours TV (29.9%) vs < half an hour (17.5%)** | **>4 hours on comp/games found easy but not strongly dose related** |
| Relaxing/ stressful | **Relaxing had strong intentions (89.2% relaxing vs 56.6% stressful)** | **Adequate exercise found relaxing (35.5% relaxing vs 3% stressful)** | **Stressful; watched >4 hours TV (12.6%) vs < half an hour (4.7%)** |  |
| Not enjoyable/enjoyable | **Enjoyable had strong intentions (91.1% enjoyable vs 32.1% not enjoyable)** | **Adequate exercise found enjoyable (51.0% enjoyable vs 1.6% not enjoyable)** | **Not enjoyable; watched >4 hours TV (14.6%) vs < half an hour (3.0%)** |  |
| Unhealthy/healthy | **Healthy had strong intentions (80.4 healthy % vs 31.3% unhealthy)** | **Adequate exercise found healthy (79.5% healthy vs 0.3% unhealthy)** | All thought -healthy |  |
| **PA SUBJECTIVE NORM** (Pleasing others) |  |  |  |  |
| **PA PBC (Control over their behaviour)** | **High confidence had strong intentions (71.5% HC vs 62.2%LC)** | **Adequate exercise had high confidence (66.8% HC vs 1.1% LC)** | **No confidence; watched >4 hours TV (20.6% ) vs < half an hour (9.6%)** | **Association did not have strong trend** |
|  |  |  |  |  |
| **PA INTENTION** |  | **Strong intention did adequate exercise (83.3% ) vs inadequate exercise (57.3%)** | **No intention watched >4 hours TV (10.3%) vs < half an hour (1.5%)** | **Strong intention < 4hours on comp/games (65.5%) vs > 4hours (59.2%)** |

Significant associations are **BOLDED**; PBC: Perceived Behavioural Control; HC: High confidence; LC: Low confidence; SI: Strong Intention; LI: Low intention
