# Supplementary material for: Physical activity attitudes, intentions and behaviour among 18–25 year olds: A mixed method study
Source: BMC Public Health. 2012 Aug 10;12:640. doi: 10.1186/1471-2458-12-640 (PMC3490897; doi:10.1186/1471-2458-12-640)
Supplement: Additional file 4 — Association between physical activity behaviour and BMI. Association between the three physical activity behaviours and BMI. [file 1471-2458-12-640-S4.doc]

Additional file 4: Association between physical activity behaviour and BMI

|  | ***BMI CATEGORIES*** | | | | |
| --- | --- | --- | --- | --- | --- |
| ***PHYSICAL ACTIVITY*** | **Underweight** | **Normal** | **Overweight** | **Obese** | **Significance**  **p-value†** |
| **Exercise**  Adequate  Inadequate | 124 (36.8%)  253 (29.1%) | 160 (47.5%)  387 (44.6%) | 44 (13.1%)  150 (17.3%) | 9 (2.7%)  78 (9.0%) | <0.001 |
| **TV watching**  < Half an hour  1 - 4 hours a day  > 4 hours a day | 122 (31.9%)  227 (30.5%)  29 (34.5%) | 179 (46.7%)  344 (46.2%)  24 (28.6%) | 60 (15.7%)  119 (16.0%)  19 (22.6%) | 22 (5.7%)  55 (7.4%)  12 (14.3%) | 0.020 |
| **Computer/games**  < Half an hour  1 - 4 hours a day  > 4 hours a day | 180 (35.4%)  156 (29.2%)  42 (25.5%) | 228 (44.8%)  246 (46.1%)  71 (43.0%) | 67 (13.2%)  96 (18.0%)  34 (20.6%) | 34 (6.7%)  36 (6.7%)  18 (10.9%) | 0.022 |

† All P values were based on Pearson Chi-square test unless otherwise highlighted
