# Supplementary material for: Physical activity attitudes, intentions and behaviour among 18–25 year olds: A mixed method study
Source: BMC Public Health. 2012 Aug 10;12:640. doi: 10.1186/1471-2458-12-640 (PMC3490897; doi:10.1186/1471-2458-12-640)
Supplement: Additional file 5 — Physical activity intention model. Physical activity intention model (Stage 1). [file 1471-2458-12-640-S5.doc]

Additional file 5: Physical activity Intention model (Stage 1)

**Stage1:**  **PA Intention LOGMOD agree (ref) VS Not agree (Logistic regression)**

by Attitudes + Subjective Norm + Intention + Demographics

| **Demographics** | **Attitudes** | **Subjective Norm** | **Perceived Behavioural Control** |
| --- | --- | --- | --- |
| Age groups | PA attitude difficult ‡† | PA Subjective Norm | PA PBC ‡† |
| Gender | PA attitude relax |  |  |
| Employment status‡ † | PA attitude enjoy ‡† |  |  |
| Level of education | PA attitude healthy ‡† |  |  |
| Study subject |  |  |  |
| Quantity of smoking |  |  |  |
| Living arrangement |  |  |  |
| Alcohol consumption |  |  |  |
| BMI categories |  |  |  |
| n=1152 Nag­ R^2^= .169 | n=1127 Nag­ R^2^= 0.353 | Not sig | n=1146 Nag­ R^2^= .028 |
| Combined PA intention Model n= 1122 Nag­ R^2^=0.552^a^; | | | |

‡ Significant p<0.05 in each block model

† Significant p<0.01 in the combined PA intention model

PA: Physical Activity; SN: Subjective Norm; PBC: Perceived Behavioural Control

Nag­ R^2^: Nagelkerke R squared – pseudo measure of fit

^a^Estimation terminated at iteration number 20 because maximum iterations has been reached. Final solution cannot be found.
