# Supplementary material for: Physical activity attitudes, intentions and behaviour among 18–25 year olds: A mixed method study
Source: BMC Public Health. 2012 Aug 10;12:640. doi: 10.1186/1471-2458-12-640 (PMC3490897; doi:10.1186/1471-2458-12-640)
Supplement: Additional file 6 — Physical activity behaviour model. Physical activity behaviour model (Stage 2 and 3). [file 1471-2458-12-640-S6.doc]

Additional file 6: Physical activity behaviour model (Stage 2 and 3)

**Stage 2 and 3: Full Physical Activity Behaviour Model by**

Demographics (significant in behaviour and intention models) + Intention + Attitudes (sig in behaviour model) + Weight perception + Intention + Barriers and Facilitators from Behaviour model

1. **Active Exercise** – Logistic regression Forward Stepwise: Do enough (ref) VS don’t do enough

| **Stages** | **Demographics** | **Attitudes** | **Subjective Norm** | **PBC** | **Intentions** | **Barriers** | **Facilitators** |
| --- | --- | --- | --- | --- | --- | --- | --- |
|  | Age groups | PA attitude difficult **§**†¶ ‡ | PA Subjective Norm | PA PBC **§**†¶ ‡ | PA intention **§**¶ | PA with opposite sex | To Win **§**¶ |
|  | Gender **§**¶ | PA attitude relax |  |  |  | PA as competition | Improve appearance |
|  | Employment status †¶ | PA attitude enjoy † ¶ |  |  |  | Lack of privacy | To be healthy |
|  | Level of education | PA attitude healthy †¶ |  |  |  | Lack of money | To relax |
|  | Study subject |  |  |  |  | Lack of information |  |
|  | Quantity of smoking | Weight Perception ^a^ ¶ |  |  |  | Disability |  |
|  | Living arrangement |  |  |  |  | Already do enough exercise **§**¶ ‡ |  |
|  | Alcohol consumption |  |  |  |  | Lack of choice **§**¶ |  |
|  | BMI categories **§**¶ |  |  |  |  | Lack of facilities |  |
|  |  |  |  |  |  | Bad weather |  |
|  |  |  |  |  |  | Lack of company |  |
|  |  |  |  |  |  | Lack of time **§**¶ |  |
| Stage 2 | n=1179  Nag R^2^= 0.062 | n=1290  Nag R^2^= 0.445 | Not sig | n=1293  Nag R^2^= 0.329 | n=1147  Nag R^2^ =0.057 | n=1265  Nag R^2^= 0.150 | n=1283  Nag R^2^= 0.027 |
| Stage 3 | Full Behaviour (Activity) Model n=1260 Nag R^2^= 0.523 | | | | | | |

**§** Significant (p<0.05) within each behaviour block model (Demographics, Attitudes, SN, PBC, Intention, Barriers, Facilitators)

† Significant at p<0.05 from combined intention model

¶ Included for full behaviour model

‡ Significant at p<0.05 for full behaviour model

PA: Physical Activity; SN: Subjective Norm; PBC: Perceived Behavioural Control

Nag­ R^2^: Nagelkerke R squared – pseudo measure of fit

^a^Weight Perception, derived from the data. Considered to be an attitude but as not part of the theoretical construct. Only included at the full behaviour model stage

1. **Sedentary TV** – Logistic regression (stepwise selection): <4hr (ref) VS >4 hrs

| **Stages** | **Demographics** | **Attitudes** | **Subjective Norm** | **PBC** | **Intentions** | **Barriers** | **Facilitators** |
| --- | --- | --- | --- | --- | --- | --- | --- |
|  | Age groups | PA attitude difficult**§**†¶ | PA Subjective Norm | PA PBC †¶ | PA intention **§**¶‡ | PA with opposite sex | To Win |
|  | Gender | PA attitude relax |  |  |  | PA as competition | Improve appearance |
|  | Employment status **§**†¶ | PA attitude enjoy **§**†¶ |  |  |  | Lack of privacy | To be healthy |
|  | Level of education | PA attitude healthy†¶ |  |  |  | Lack of money | To relax **§**¶ |
|  | Study subject |  |  |  |  | Lack of information |  |
|  | Quantity of smoking | Weight Perception ^a^ ¶ |  |  |  | Disability **§**¶ |  |
|  | Living arrangement |  |  |  |  | Already do enough exercise |  |
|  | Alcohol consumption |  |  |  |  | Lack of choice **§**¶‡ |  |
|  | BMI categories **§**¶‡ |  |  |  |  | Lack of facilities |  |
|  |  |  |  |  |  | Bad weather **§**¶ |  |
|  |  |  |  |  |  | Lack of company |  |
|  |  |  |  |  |  | Lack of time |  |
| Stage 2 | n=1211  Nag R^2^= 0.046 | n=1281  Nag R^2^= 0.074 | Not sig | Not sig | n=1151  Nag R^2^=0.030 | n=1142  Nag R^2^= 0.056 | n=1282  Nag R^2^= 0.020 |
| Stage 3 | Full Behaviour (Sedentary TV) Model n=1051 Nag R^2=^ 0.081 | | | | | | |

**§** Significant (p<0.05) within each behaviour block model (Demographics, Attitudes, SN, PBC, Intention, Barriers, Facilitators)

† Significant at p<0.05 from combined intention model

¶ Included for full behaviour model

‡ Significant at p<0.05 for full behaviour model

PA: Physical Activity; SN: Subjective Norm; PBC: Perceived Behavioural Control

Nag­ R^2^: Nagelkerke R squared – pseudo measure of fit

^a^Weight Perception, derived from the data. Considered to be an attitude but as not part of the theoretical construct. Only included at the full behaviour model stage

1. **Sedentary Computer/games** – Logistic regression (stepwise selection): <4hr (ref) VS >4 hrs

| **Stages** | **Demographics** | **Attitudes** | **Subjective Norm** | **PBC** | **Intentions** | **Barriers** | **Facilitators** |
| --- | --- | --- | --- | --- | --- | --- | --- |
|  | Age groups | PA attitude difficult †¶ | PA Subjective Norm | PA PBC †¶ | PA intention | PA with opposite sex | To Win |
|  | Gender **§**¶ ‡ | PA attitude relax |  |  |  | PA as competition | To improve appearance |
|  | Employment status †¶ | PA attitude enjoy **§**†¶ |  |  |  | Lack of privacy | To be healthy |
|  | Level of education **§**¶ ‡ | PA attitude healthy †¶ |  |  |  | Lack of money | To relax |
|  | Study subject |  |  |  |  | Lack of information |  |
|  | Quantity of smoking | Weight Perception ^a^ ¶ |  |  |  | Disability **§**¶‡ |  |
|  | Living arrangement |  |  |  |  | Already do enough exercise |  |
|  | Alcohol consumption |  |  |  |  | Lack of choice |  |
|  | BMI categories |  |  |  |  | Lack of facilities |  |
|  |  |  |  |  |  | Bad weather |  |
|  |  |  |  |  |  | Lack of company |  |
|  |  |  |  |  |  | Lack of Time |  |
| Stage 2 | n=1243  Nag R^2^= 0.058 | n=1287  Nag R^2^= 0.014 | Not sig | Not sig | Not sig | n=1284  Nag R^2^= 0.007 | Not sig |
| Stage 3 | Full Behaviour (Sedentary Computer) Model Nag R^2^=0.065, n=1222 | | | | | | |

**§** Significant (p<0.05) within each behaviour block model (Demographics, Attitudes, SN, PBC, Intention, Barriers, Facilitators)

† Significant at p<0.05 from combined intention model

¶ Included for full behaviour model

‡ Significant at p<0.05 for full behaviour model

PA: Physical Activity; SN: Subjective Norm; PBC: Perceived Behavioural Control

Nag­ R^2^: Nagelkerke R squared – pseudo measure of fit

^a^Weight Perception, derived from the data. Considered to be an attitude but as not part of the theoretical construct. Only included at the full behaviour model stage
