# Supplementary material for: Physical activity attitudes, intentions and behaviour among 18–25 year olds: A mixed method study
Source: BMC Public Health. 2012 Aug 10;12:640. doi: 10.1186/1471-2458-12-640 (PMC3490897; doi:10.1186/1471-2458-12-640)
Supplement: Additional file 7 — Results of the focus groups. [file 1471-2458-12-640-S7.doc]

Additional file 7: Results of the focus groups

| **Theme** | **Sub-theme** | **Quotes** |
| --- | --- | --- |
| 1. PA behaviour | Present behaviour | *1a: “I cycle quite a bit, like basically where ever I go I use the bike instead of the bus. I play squash once, two, twice a week. I dance ballet. Anything else? Oh yeah, during the summer I do kite surfing.” (University; 20-24 age group)*  *1b: “I do quite a lot too. Running and hiking and stuff.* *I live so far away if I then go mountain biking I just take it up to the Highlands.” (College; 18-21 age group)*  *1c: “Asda I work 15, 16 hour days. We used to walk to work, walk all day, and then walk home at 10 o’clock at night. Wondered why I was tired cause I had to get up at four in morning the next day.” (Inner city; 18-21 age group)*  *1d:“Aye, never sit, never get a seat. With my kids. Up and down and that.” (Young mothers; 21-24 age group)* |
|  | Past behaviour | *1e: “I used to cycle a lot. And sometimes I went to rugby classes. It was fun. Swimming as well. So much more active than here.” (University; 18-19 age group)*  *1f: “Used to play foot ball. I used to do running.” (Shire –Not in education, employment or training-NEET; 18-19 age group)* |
| 2. Influences on PA behaviour | Parents/partners (+ve) | *2a:: “But you’re like “I can’t really be bothered”. And my mum’s like “well I’m going, so I’ll go with you”. It’s like “OK I actually will go then”. It’s more encouraging to do more.” (University; 18-19 age group)* |
|  | Necessity (+ve) | *2b: “If customers bug me for shoes I can’t be bothered waiting for the lifts so I just take the stairs. I get that 24/7.” (Inner city-working; 18-21 age group)* |
|  | Reasons for doing exercise (+ve) | *2c: “Makes me feel really good. Especially if, I don’t know, I just feel really relaxed afterwards. ” (College; 18-21 age group)*  *2d: “I have to exercise because otherwise I feel bad...So if I don’t exercise I feel that I’m getting depressed. Not really depressed depressed. But I start like feeling bad, my whole body.” (University; 18-19 age group)*  *2e: “Well I just love it. Concerning cycling I love it.” (College; 18-21 age group)*  *2f: “I don’t know. Feel a bit confident.. to do more stuff....Just feeling happy ..you know.” (Shire-NEET group; 18-19 age group)* |
|  | Reasons for not doing exercise (-ve) | *2g: “And then I just got into the whole student lifestyle, boozing up and all that and that, really cut me back. That’s probably what did it. Cause you, the first few months you try and settle in. You just go out. You got no push to do anything. You know.” (University; 18-19 age group)*  *2h: “Don’t really want to go swimming on own either. Looking like a plug, if you walked into a swimming pool yourself.” (Inner city-working; 18-21 age group)*  *2i: “The gym’s are so expensive as well. It’s £32 a month. It’s not that much but being a student it is a chunk coming out.” (College; 20 -22 age group)*  *2j: “I’ve been to this gym. Here. And it’s just not suited for the amount of people using it. It’s just too small, the actual...... because it is just so crowded it is not a good environment to be in.” (University; 18-19 age group)*  *2k: “They’ve got a lot more facilities for like the younger generation. But nothing. They don’t. They tend to forget about us. I don’t think there is enough for people our age to do.” (Inner city-working; 18-21 age group)*  *2l: “I think it’s quite intimidating. I would have thought about joining some societies. But then, I thought like “am I actually good enough to do that?” Like I’d hate to just go along and everyone was really really good. .. Like I’d rather do something by myself …Instead of like a society or a team sport that’s competitive.” (University; 18-19 age group)*  *2m: “I’m quite lazy. I am properly lazy, believe it or not. Well like I said, the Chinese is across the road from my house. Last night I could easily have walked down the stairs and across but nup, I phoned them and got it home delivered to me.... that is lazy.” (Inner city-working; 18-21 age group)*  *2n: “Because I think if I could be bothered, I could get up and do something myself but I just can’t be bothered. (University; 18-19 age group)*  *2o: “If they’re at night as well. Cause I don’t particularly like wandering around in the dark. Even if I got a bus back I don’t like hanging around waiting for buses.” (University; 18-19 age group)* |
|  | Assumptions and preferences | *2p: “I don’t really do a lot of exercise. Every now and again I consider the gym. But I’ve never been to the gym. ........ As I say I don’t really do much exercise. It’s strange I walk everywhere. But you’re right (T05’s comment) it’s not really exercise its just sort of a practical thing.” (University; 20-24 age group)*  *2q: “Making up all the tables is working is it..?” (Shire-NEET group; 18-19 age group)*  *2r: “It doesn’t make sense that I’m so concerned with healthy eating and stuff and not with the exercise. …But is a bit of an effort. I wanted to eat healthy in a way that I don’t really want to exercise. So. Making the effort is a bit. It feels like more of an effort.” (University; 20-24 age group)* |
|  | Subjective norm | *2s: “I’m just like…..I think I get this image of this supermodel who has to take really good care of her diet and her exercise. And I’m just, this is not my life. I like to do things for the purpose of doing them, enjoying them. Not for social constraints or whatever...” (University; 20-24 age group)* |
| 3. Attitudes towards Physical activity | On exercise and others PA behaviour | *3a: “If I walked there and back then I wouldn’t actually want to do any exercise in the middle. So the other option is to get a bus, which obviously costs more money, and you’re cutting out exercise just to do a different type of exercise. Which I’d probably actually, just go to the gym and go for like 15 minutes and then give up a because I’d be too tired.” (University; 18-19 age group)*  *3b: “…Her idea of a healthy diet is purely calorie based. So as long as she doesn’t eat over 1500 or 2000 calories, if its chocolate etc. She knows that fruit and vegetables are healthy.” (University; 20-24 age group)*  *3c: “Well they just hate anyone who does exercise. They think they’re jocks. No. No, they wouldn’t have a go at me if I did exercise. …..So we can’t really talk about sports that much.” (University; 20-24 age group)*  *3d: “(mum) ...she’s like, “we can join together”. It’s like. Just you go. Go yourself.” (Inner city-working; 18-21 age group)* |
|  | About concern for future health | *3e: “I don’t tend to think into the future. I’m always aware in the back of my mind that especially as I get older some form of exercise would be a good idea. It is good for you. I’m very aware of that.” (University; 20-24 age group)*  *3f: “Not worried..na” (Shire-NEET group; 18-19 age group)* |
|  | On health promotion messages | *3g: I see fliers everywhere. Maybe it is the message on these fliers that at least doesn’t stick with me. Because it’s like “be healthy, do sports”. I know that. Of course, of course…But it’s not the right button to press in my case….Hey, squash is so fun. It’s so fast, it gives you a kick, its better than taking drugs. And I would be like, oh really, fab.” (University; 20-24 age group)* |
| 4. Behaviour change | Motivators for behaviour change | *4a: “If there’s someone else telling you to do it. If there’s like one voice in your head saying “I can’t be bothered to go but I know I should”. But you’ve got an outside voice saying “yeah I’ll go with you”. You’re more likely to go.” (University; 18-19 age group)*  *4b: “If they wouldn’t have been so competitive or whatever, maybe I would have joined them any how.” (University; 18-19 age group)*  *4c: “You know how they give you points and things. And you’re going there every week…... That’s a goal for you isn’t it so you would stick to it………………I think that would help me doing something like that.” (Young mothers; 21-24 age group)* |
|  | Intention and perceived behavioural control | *4d: “The female friends I have in the college are always going on about xxxx and not exercising enough and all that stuff.. Doesn't turn to action.” (College; 18-21 age group)*  *4e: “That is my New Year’s resolution is to eat better...exercise more..cut down on smoking. Don’t know what will happen.” (Shire-NEET group; 18-19 age group)* |
| 5. Knowledge | On physical activity | *5a: “If you don’t exercise, you’re going to get unhealthy. You always read in magazines and see on TV. If you don’t do exercise you’re going to get heart disease and die when you’re 50.” (University; 18-19 age group)*  *5b: “I will get a lot bigger than I am. I will be massive in next 10 years like.. If I don’t start doing any exercise..I would end up massive.”* *(Shire-NEET group; 18-19 age group)* |
|  | Influencing others | *5c: “I drag them (friends) up the stairs every day. They always “I’d really like to go with you”. But they never do. And if they do they just get really tired and then it was such a big effort. It doesn’t really inspire them to go again really.” (College; 18-21 age group)*  *5d: “ I say ‘Do you want to go swimming??’. Everybody (friends) is like, ‘no’...”(Inner city-working; 18-21 age group)* |
